# Brain structural alterations in young women with premature ovarian insufficiency: Implications for dementia risk

**DOI:** 10.1002/alz.70111

**Published:** 2025-03-27

**Authors:** Shuang Yuan, Yuchen Gong, Yu Zhang, Wenjiao Cao, Liutong Wei, Taotao Sun, Junyan Sun, Lulu Wang, Qiuwan Zhang, Qian Wang, Yu Wei, Zhaoxia Qian, Puming Zhang, Dongmei Lai

**Affiliations:** ^1^ The International Peace Maternity and Child Health Hospital, School of Medicine Shanghai Jiao Tong University Shanghai China; ^2^ Shanghai Key Laboratory of Embryo Original Diseases Shanghai China; ^3^ School of Biomedical Engineering Shanghai Jiao Tong University Shanghai China

**Keywords:** Alzheimer's disease, brain morphology, case–control study, dementia, premature ovarian insufficiency, structural network

## Abstract

**INTRODUCTION:**

Premature ovarian insufficiency (POI), marked by ovarian function loss before age 40, is linked to a higher risk of dementia, including Alzheimer's disease (AD). However, the associated brain structural changes remain poorly understood.

**METHODS:**

We analyzed T1‐weighted and diffusion tensor imaging in 33 idiopathic POI women and 51 healthy controls, using voxel‐based, surface‐based morphometry, and network analyses to assess gray matter volume (GMV), cortical thickness, and brain connectivity.

**RESULTS:**

Women with POI showed significant GMV and cortical thickness reductions in the frontal, parietal, and temporal regions (*p* < 0.05), alongside impaired connectivity with key regions such as the hippocampus, thalamus, and amygdala (*p* < 0.05). Younger POI subgroups exhibited changes in more widespread brain regions. In additionally, notable atrophy was observed in specific hippocampal and thalamic subregions in POI (*p* < 0.05).

**DISCUSSION:**

This preliminary study suggests early neurodegenerative patterns in POI, potentially contributing to dementia risk. Further research is needed to explore the underlying mechanisms and potential interventions.

**Highlights:**

- We evaluated brain structural changes in participants with idiopathic premature ovarian insufficiency (POI).
- The observed brain alterations in POI participants closely resemble those seen in early dementia, including regions specifically associated with Alzheimer's disease (AD).
- These findings highlight the critical need for early interventions to reduce the long‐term risks of cognitive impairment and dementia in women with POI.

## 1 BACKGROUND

Dementia is a broad term encompassing a range of symptoms characterized by impairments in memory, language, problem‐solving, and other cognitive functions that significantly interfere with daily activities.^1^ Among its various forms, Alzheimer's disease (AD) is the most prevalent, marked by progressive cognitive decline and neurodegeneration. Epidemiological studies reveal a significant sex disparity in AD incidence, with a male‐to‐female ratio of 1:2 after the age of 50, accounting for ≈70% of cases.^2^ In addition to AD, other forms of dementia, including vascular dementia, Lewy body dementia, and frontotemporal dementia, also exhibit a higher prevalence in women.^1^

Menopause, typically occurring between the ages of 45 and 55, represents a natural biological transition marked by the cessation of ovarian function and a decline in circulating estrogen levels. The menopausal transition, or perimenopause, can span several years and often significantly affects physical, emotional, mental, and social well‐being due to menopause‐related symptoms. However, women who experience menopause before the age of 40 are diagnosed with premature ovarian insufficiency (POI), a condition affecting ≈3.5% of the female population, with its incidence rising in recent years.^3^, ^4^ Beyond its reproductive implications, POI is strongly associated with an elevated risk of cardiovascular disease and stroke, both of which are closely linked to brain structural changes and cognitive decline.^4^ The etiology of POI is multifactorial, involving genetic mutations, autoimmune disorders, and iatrogenic factors. Notably, in more than 50% of cases, the underlying cause remains unidentified and is classified as idiopathic POI.^5^

Research indicates that the pathological changes underlying AD, including the deposition of amyloid beta (Aβ) protein, tau tangles, and chronic glial cell proliferation, may begin 10–20 years before the onset of clinical symptoms, overlapping with the perimenopausal period (ages 40–60).^6^ Consequently, perimenopause represents a critical window for early intervention in AD. Studies have demonstrated that the earliest brain structural changes in AD predominantly affect the hippocampus and entorhinal cortex‐key regions essential for memory and learning. Hippocampal atrophy can initiate 10–20 years prior to the manifestation of clinical symptoms, such as memory impairment.^7^ Magnetic resonance imaging (MRI) studies further corroborate these findings, revealing early reductions in gray matter volume in regions like the temporal and parietal lobes, which are crucial for memory and spatial navigation.^8^

Estrogen plays a vital neuroprotective role by enhancing synaptic plasticity, promoting the production of memory‐related neurotransmitters, facilitating neuronal repair through cytokines, and reducing Aβ production.^9^ Estrogen deficiency has been associated with impaired language fluency, deficits in visual memory, and a 30% increased risk of cognitive decline in later life.^10^, ^11^ Furthermore, prolonged hormonal imbalance significantly exacerbates the risk of neurodegeneration, particularly AD, and is strongly linked to a higher incidence of dementia—especially vascular dementia—and cardiovascular diseases, including strokes.^12^ Given these findings, we propose that POI, due to its earlier onset and prolonged estrogen deficiency, may lead to earlier brain structural changes and an elevated risk of cardiovascular and neurological disorders compared to natural menopause.

Despite progress in early AD detection, identifying preclinical brain changes remains challenging due to the subtle nature of early pathological alterations. However, advancements in MRI technology now enable the detection of brain structural changes at earlier stages. Studies have shown that women who undergo bilateral oophorectomy before menopause exhibit reduced white matter integrity across multiple brain regions later in life, highlighting potential long‐term neurostructural consequences.^13^ Nevertheless, evidence connecting brain structural changes in individuals with idiopathic POI to dementia remains scarce. To address this gap, the present study employs advanced neuroimaging techniques to investigate brain injury progression in idiopathic POI participants. This approach aims to uncover early markers of dementia and facilitate timely assessments for at‐risk individuals.

**RESEARCH‐IN‐CONTEXT**

1. **Systematic review**: A review of the existing literature on premature ovarian insufficiency (POI) and dementia, particularly Alzheimer's disease (AD), reveals a scarcity of studies examining the neurobiological effects of POI.
2. **Interpretation**: Our study offers novel insights by identifying significant brain structural alterations in young women with POI, including reduced gray matter volume and cortical thickness in regions critical for memory and cognition, such as the hippocampus and thalamus. These findings suggest that POI may accelerate neurodegenerative processes akin to those seen in early‐stage AD, underscoring the potential impact of POI on brain health.
3. **Future directions**: This study establishes a foundation for further exploration of the neurobiological consequences of POI. Future studies should focus on investigating long‐term brain structural changes and their relationship to cognitive decline, incorporating comprehensive neuropsychological assessments to detect subtle cognitive or neurological changes, and developing early intervention strategies to reduce dementia risk in women with POI.

## 2 METHODS

### 2.1 Study participants

This study employed a hospital‐based case–control design conducted at the International Peace Maternity and Child Health Hospital (IPMCH) in Shanghai, China, from August 2022 to August 2023. A total of 33 newly diagnosed idiopathic POI participants who had not yet received hormone replacement therapy (HRT) and 51 healthy individuals undergoing routine check‐ups during the same period were recruited. For POI participants, the time between their last menstrual period and diagnosis ranged from 1 to 6 months (Figure S1). Participants were excluded if they used oral contraceptives or had chronic conditions such as hypertension, hyperlipidemia, diabetes, or cardiovascular disease. The study protocol was approved by the IPMCH Ethics Committee (GKLW2023‐007), and written informed consent was obtained after a detailed explanation of the study procedures.

Idiopathic POI was defined as oligomenorrhea or amenorrhea, accompanied by follicle‐stimulating hormone (FSH) levels exceeding 25 IU/L on two separate occasions at least 4 weeks apart, occurring before the age of 40, with no identifiable underlying cause. Exclusion criteria included: (1) abnormal chromosomes and/or Fragile X Mental Retardation 1 premutation; (2) prior exposure to iatrogenic factors such as chemotherapy, radiotherapy, or bilateral oophorectomy; (3) autoimmune disorders, including systemic lupus erythematosus, Addison's disease, or positivity for thyroid peroxidase antibody (TPO‐Ab) and/or adrenocortical antibody (ACA); and (4) a diagnosis of polycystic ovary syndrome (PCOS).

The control group comprised individuals with normal FSH levels (<10 IU/L), regular menstrual cycles (25–35 days), and no use of hormonal contraceptives within the preceding 6 months. Exclusion criteria for the control group included: (1) a diagnosis of PCOS; (2) prior exposure to iatrogenic factors such as chemotherapy, radiotherapy, or bilateral oophorectomy; and (3) autoimmune disorders, including systemic lupus erythematosus, Addison's disease, or positivity for TPO‐Ab and/or ACA.

### 2.2 Data collection

Information on age, age at menarche, reproductive history, smoking status, and alcohol consumption was collected through face‐to‐face interviews conducted by trained clinicians using a standardized questionnaire. Professional clinic staff measured weight and standing height, which were used to calculate body mass index (BMI) as weight (kg) divided by the square of height (m^2^). Additional medical information, including prior chemotherapy, radiotherapy, surgeries, and gynecological examinations, was obtained from medical records.

### 2.3 Sample collection and hormone testing

Blood samples were collected from control participants between Days 2 and 4 of their menstrual cycle, whereas samples from POI participants were obtained at a random time point.^4^ All samples were drawn from the median cubital vein. Serum was separated for the measurement of alanine aminotransferase (ALT), aspartate aminotransferase (AST), creatinine, FSH, luteinizing hormone (LH), estradiol (E2), testosterone (T), prolactin (PRL), and anti‐Müllerian hormone (AMH) levels. ALT, AST, creatinine, FSH, LH, E2, T, and PRL concentrations were assessed using a chemiluminescent assay (Beckman Coulter, USA), whereas AMH levels were measured using an enzyme‐linked immunoassay (ELISA) kit (Roche).

### 2.4 The assessment of cognition, mood, and menopause systems

Trained staff conducted face‐to‐face interviews to assess cognition in both POI participants and healthy controls using the Mini‐Mental State Examination (MMSE).^14^ Mood assessments were also performed during this session, including the Patient Health Questionnaire‐9 (PHQ‐9) to evaluate depressive symptoms and the Self‐Rating Anxiety Scale (SAS) to assess anxiety symptoms.^15^, ^16^ Higher scores on these measures indicated worse mood. In addition, all participants completed the Chinese version of the Menopause‐Specific Quality of Life Questionnaire (MENQOL) (Figure S2).^17^ This validated tool, designed specifically for Chinese women, evaluated menopausal symptoms across four domains: vasomotor, physical, psychosocial, and sexual. Each item was scored on a scale from 1 to 8, with higher scores indicating greater symptom severity and lower quality of life. Domain‐specific symptom burden was quantified by calculating the average score for each category.

### 2.5 Voxel‐based and surface‐based morphometry analysis based on T1‐weighted images

We analyzed cerebral MR images from 33 women with idiopathic POI (16 ages 20–35 years and 15 ages 36–40 years) and 51 healthy controls (27 ages 20–35 years and 24 ages 36–40 years). Imaging was performed on a 1.5‐Tesla MRI system equipped with a 20‐channel head‐neck coil (Aera, Siemens, Erlangen, Germany). T1‐weighted images were acquired in the sagittal plane using a gradient‐recalled echo (GRE) sequence with the following parameters: repetition time (TR) = 10 ms, echo time (TE) = 4.76 ms, slice thickness = 1 mm, 192 slices, flip angle = 20°, matrix = 256 × 256, and field of view (FOV) = 250 × 250 mm^2^. Diffusion tensor imaging (DTI) was performed using a two‐dimensional (2D) echo‐planar imaging (EPI) sequence with the following parameters: TR = 3500 ms, TE = 83 ms, slice thickness = 5 mm, gap = 6.5 mm, flip angle = 90°, matrix = 128 × 128, FOV = 230 × 230 mm^2^, with 20 diffusion‐encoding gradient directions (b‐value = 1000 s/mm^2^) and 12 non‐diffusion‐weighted images (b = 0 s/mm^2^).

T1‐weighted images were preprocessed using the Statistical Parametric Mapping 12 toolbox (SPM12, www.fil.ion.ucl.ac.uk/spm/software/spm12/), including co‐registration to the Montreal Neurological Institute (MNI) space, segmentation of gray and white matter, and smoothing with an 8‐mm full‐width at half‐maximum (FWHM) Gaussian kernel. Volume‐based morphometry (VBM) features, such as global and regional gray matter volume (GMV) and white matter volume (WMV), were extracted. Regions of interest (ROIs) were defined using the Anatomical Automatic Labeling (AAL) atlas, which includes 90 cerebral regions (Table S1).^18^ In addition, subregions in the hippocampus and thalamus were analyzed using the Computational Brain Anatomy (CoBrA)^19^ and the human Brainnetome Atlas (BN246),^20^ respectively. White matter masks were created for subsequent diffusion analysis.

Surface‐based morphometry (SBM) features were computed using the Computational Anatomy Toolbox 12 (CAT12), assessing cortical thickness, sulcal depth, gyrification index, and fractal dimension.^21^, ^22^, ^23^, ^24^ The Desikan‐Killiany (DK40) atlas was co‐registered to individual surface models for regional SBM feature extraction.^25^

### 2.6 DTI preprocessing and structural network construction

DTI data preprocessing was performed using the MRtrix3 toolbox, following a standardized workflow that included denoising, removal of Gibbs artifacts, eddy‐current correction, and registration to the MNI space.^26^ White matter fiber tracking was conducted by generating 10 million probabilistic streamlines using the Second‐order Integration over Fiber Orientation Distributions (iFOD2) algorithm (step size = 0.2 mm, length = 10–400 mm).^27^ Tractography‐based individual structural networks were parcellated into 90 cerebral regions using the AAL atlas. The edges of structural networks were defined as the number of streamlines between pairs of nodes and normalized by the maximum connectivity.^28^

Global graph‐theoretical features of the weighted network were calculated, including the mean clustering coefficient (MCC), characteristic path length (CPL), and small‐worldness (SW).^29^ MCC quantifies the likelihood of connectivity among a node's neighbors, whereas CPL measures the shortest path between two nodes. A small‐world network is characterized by a high MCC and a CPL approximately equivalent to that of matched random networks, with SW >1.^30^ Individual MCC and CPL were normalized by their respective means from 100 random networks generated through rewiring‐based randomization.^31^ SW was then computed as the ratio of MCC to CPL.

Nodal features, including nodal degree, clustering coefficient, and local efficiency, were also analyzed.^32^, ^33^ Nodal degree represents the sum of connections with all other nodes in the network. The clustering coefficient evaluates the connectivity between a node and its nearest neighbors, whereas local efficiency measures the efficiency of communication among these neighbors when the node is removed.^33^, ^34^

SPM, CAT12, and graph‐theoretical analyses were implemented in MATLAB R2021b (MathWorks, Natick, MA, USA), whereas MRtrix3 operations were performed in an Ubuntu 18.04 environment.

### 2.7 Statistical analysis

The characteristics of participants were described using counts and percentages for categorical variables. The chi‐square test was employed to compare the categorical sociodemographic characteristics between POI and control groups. The Shapiro–Wilk test was applied to assess the normality of data distributions. For normally distributed data, the Student's *t*‐test was used to evaluate significant differences between groups. When data did not follow a normal distribution, the nonparametric Mann‐Whitney *U* test was performed. Pearson's correlation was applied to examine associations between the duration from the last menses to the POI diagnosis and changes in GMV and cortical thickness. Hormonal levels (FSH, LH, E2, T, PRL, and AMH) were each tested in relation to GMV and cortical thickness using linear regression models. Covariates in these models included age, BMI, parity, smoking status, and alcohol consumption. Due to the exploratory nature of the analyses, no adjustments were made for multiple comparisons.^13^

Data analysis was carried out using SPSS 25.0 (IBM, Armonk, NY, USA). Statistical significance was defined as *p*‐values < 0.01 for structural connectivity differences and *p*‐values < 0.05 for other comparisons. Unless otherwise indicated, all statistical tests were two‐tailed, with one‐tailed tests explicitly marked.

## 3 RESULTS

### 3.1 Characteristics of participants

The primary demographic and clinical characteristics of the study participants are presented in Table 1. A total of 84 women met the inclusion criteria, including 33 POI participants and 51 age‐matched healthy controls. Statistical analysis revealed no significant differences between the groups in terms of age, BMI, age at menarche, parity, smoking status, and alcohol consumption. Furthermore, there were no statistically significant differences in liver and kidney functions, including ALT, AST, and creatinine levels, between the two groups. To assess ovarian function, serum hormone levels were measured. Compared to the control participants, POI participants had significantly higher levels of FSH (*p* < 0.001) and LH (*p* < 0.001), whereas levels of E2 (*p* < 0.001), PRL (*p* = 0.001), and AMH (*p* < 0.001) were significantly lower in the POI group.

**TABLE 1:** Demographic and clinical characteristics of participants.

Characteristic	POI group (*n* = 33)	Control group (*n* = 51)	*p*
Age (y)	34.3 ± 5.4	32.5 ± 5.8	0.154^a^
BMI (kg/m^2^)	20.9 ± 2.4	21.2 ± 2.3	0.540^a^
Age at menarche (y)	13.2 ± 1.3	13.1 ± 1.3	0.535^a^
Parity^*^			0.871^b^
0	20 (60.6)	30 (58.8)	
≥1	13 (39.4)	21 (41.2)	
Smoking status			0.830^b^
No	32 (97.0)	49 (96.1)	
Yes	1 (3.0)	2 (3.9)	
Alcohol consumption			0.974^b^
No	15 (45.5)	23 (45.1)	
Yes	18 (54.5)	28 (54.9)	
ALT (U/L)	18.6 ± 8.2	15.3 ± 8.3	0.070^c^
AST (U/L)	19.7 ± 6.4	18.3 ± 9.7	0.390^a^
Creatinine (µmol/L)	64.2 ± 8.1	66.0 ± 11.6	0.412^a^
FSH (IU/L)	66.2 ± 26.6	6.6 ± 2.0	<0.001^c^
LH (IU/L)	34.4 ± 17.4	9.3 ± 7.2	<0.001^c^
E2 (pmol/L)	171.9 ± 252.0	354.3 ± 398.3	<0.001^c^
T (nmol/L)	1.4 ± 0.8	1.6 ± 0.5	0.108^a^
PRL (ug/L)	9.6 ± 5.5	12.8 ± 6.0	0.001^c^
AMH (ng/mL)	0.1 ± 0.0	3.9 ± 2.7	<0.001^c^

*Note*: The data are shown as mean ± SD or count (percentage).

Abbreviations: ALT, alanine aminotransferase; AMH, anti‐Mullerian hormone; AST, aspartate aminotransferase; BMI, body mass index; E2, estradiol; FSH, follicle‐stimulating hormone; LH, luteinizing hormone; POI, premature ovarian insufficiency; PRL, prolactin; T, testosterone; y, years; SD, standard deviation.

* Parity is defined as the number of pregnancies that a woman delivers.

^a^ Student's *t*‐test for independent samples.

^b^ chi‐square test.

^c^ Mann‐Whitney *U* test.

None of the participants exhibited clinically evident symptoms, such as motor dysfunction, sensory deficits, or other neurological abnormalities, as reported during the structured medical interview. In addition, MMSE scores were assessed to evaluate potential cognitive impairments, revealing no significant differences between the two groups. However, POI participants reported a significantly greater negative impact on MENQOL, particularly in vasomotor, psychosocial, physical, and sexual symptoms (*p* < 0.05) (Table S2). In addition, although depressive (PHQ‐9) and anxiety (SAS) scores were slightly higher in the POI group, these differences were not statistically significant. These findings highlight the absence of overt cognitive decline in POI participants but underscore the significant quality of life burden associated with the condition.

### 3.2 Voxel‐based morphometry and surface‐based morphometry analysis

#### 3.2.1 Brain morphological changes in POI participants at the age of 20–40 years

**FIGURE 1:**
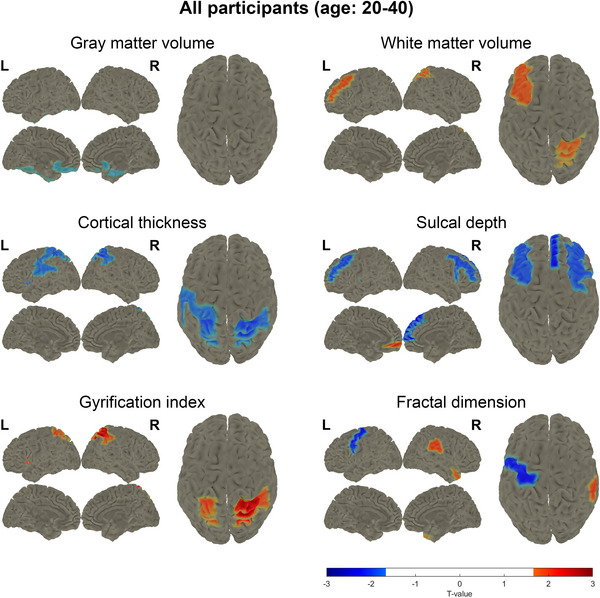
*t*‐test map of regions with significant difference between premature ovarian insufficiency (POI) participants and control group (ages 20–40 years) in gray matter volume, white matter volume, cortical thickness, sulcal depth, gyrification index, and fractal dimension. Red color means the significant increase in POI participants and blue color means the significant decrease in POI participants. L, left; R, right.

Whole‐brain data analysis revealed no significant differences in GMV, WMV, cortical thickness, sulcal depth, gyrification index, or fractal dimension between the POI group and the control group (Table 2). However, regional voxel‐wise analysis identified significant differences between the two groups. Specifically, the POI group exhibited significantly lower GMV in the bilateral olfactory cortex and the right parahippocampal gyrus, along with higher WMV in the left middle frontal gyrus and right superior parietal gyrus compared to the control group (Figure 1). In addition, the POI group showed significantly decreased cortical thickness in the bilateral parietal lobe and left insular lobe (Figure 1). Sulcal depth was significantly reduced in the frontal lobe and left transverse temporal gyrus, with the exception of increased sulcal depth in the left medial orbitofrontal region in the POI group (Figure 1). Notably, significant increases in gyrification index were observed in the parietal lobe and left insular lobe of the POI group (Figure 1). Finally, the POI group displayed significant decreases in fractal dimension in the left precentral gyrus, whereas increases were noted in the right supramarginal gyrus and right temporal pole (Figure 1). These findings suggest that, although global structural differences were not observed, POI participants exhibit distinct regional brain alterations, particularly in areas associated with memory, cognition, and sensory processing.

**TABLE 2:** Global voxel‐based morphometry and surface‐based morphometry features of POI and control groups (ages 20–40 years).

Characteristic	POI group (*n* = 33)	Control group (*n* = 51)	*p*
Gray matter volume (cm^3^)	586.957 ± 38.607	589.936 ± 51.032	0.775^a^
White matter volume (cm^3^)	393.196 ± 31.015	394.022 ± 36.793	0.915^a^
Cortical thickness (mm)	2.447 ± 0.081	2.478 ± 0.107	0.163^a^
Sulcal depth (mm)	8.086 ± 0.324	8.207 ± 0.278	0.072^a^
Gyrification index	30.935 ± 0.424	30.774 ± 0.481	0.120^a^
Fractal dimension	2.588 ± 0.019	2.589 ± 0.022	0.990^a^

*Note*: The data are shown as mean ± SD.

Abbreviation: POI, premature ovarian insufficiency; SD, standard deviation.

^a^ Student's *t*‐test for independent samples.

#### 3.2.2 Effects of onset age of POI on brain morphological changes

**FIGURE 2:**
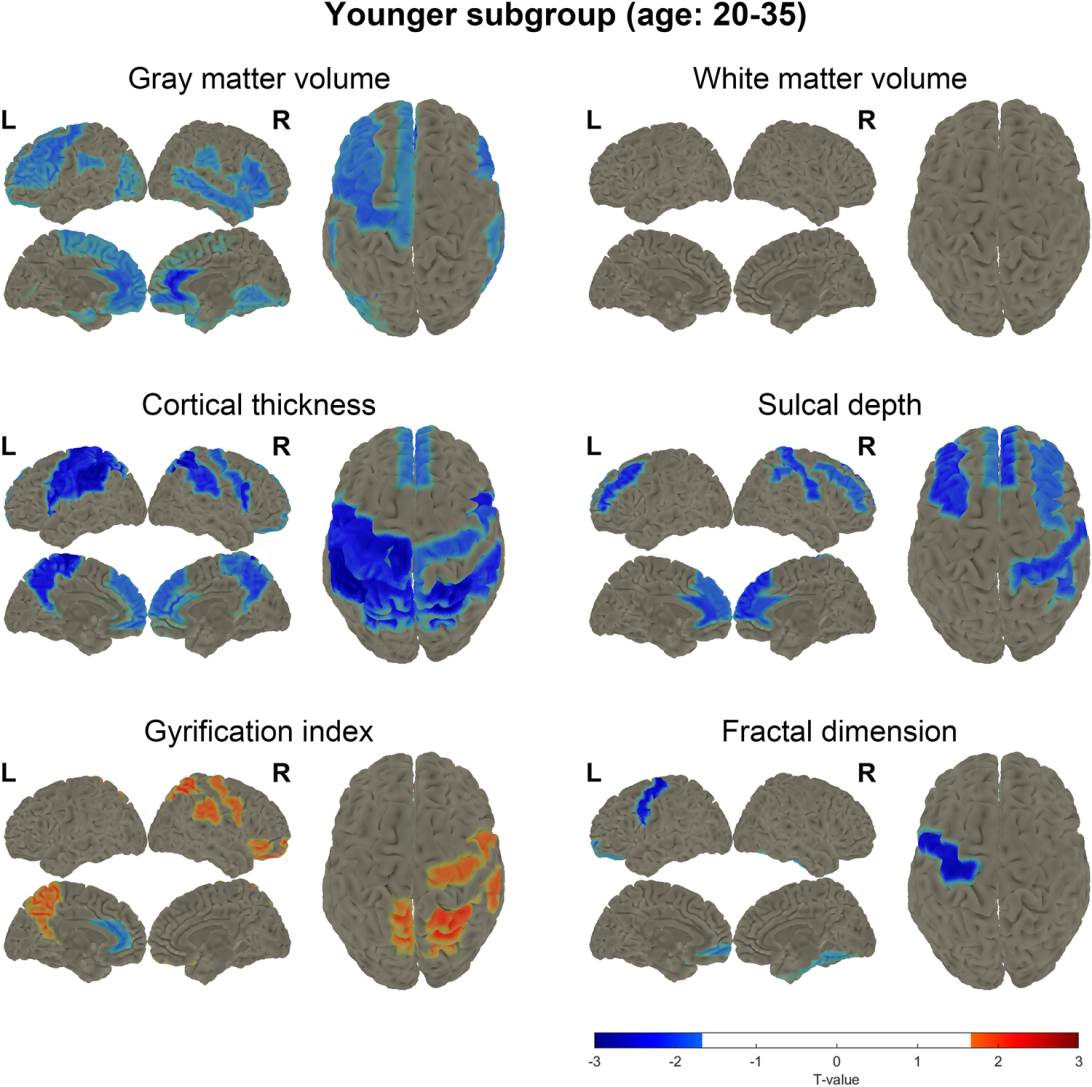
*t*‐test map of regions with significant difference between premature ovarian insufficiency (POI) and control participants of the younger subgroup (ages 20–35 years) in gray matter volume, white matter volume, cortical thickness, sulcal depth, gyrification index, and fractal dimension. Red color means the significant increase in POI participants and blue color means the significant decrease in POI participants. L, left; R, right.

**FIGURE 3:**
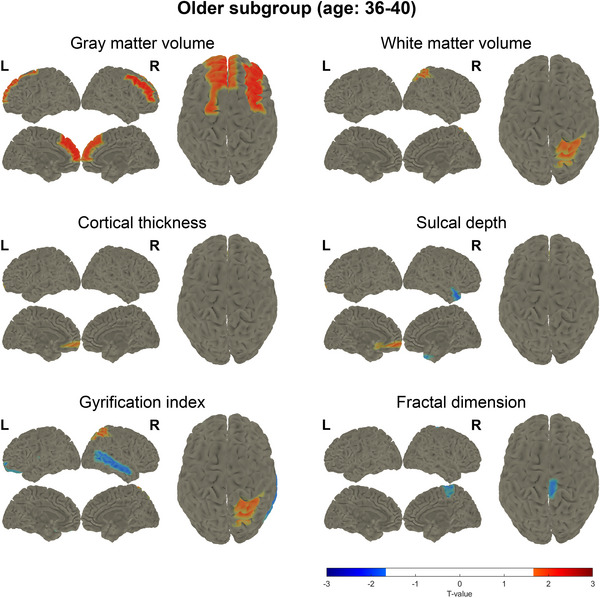
*t*‐test map of regions with significant difference between premature ovarian insufficiency (POI) and control participants of the older subgroup (ages 36–40 years) in gray matter volume, white matter volume, cortical thickness, sulcal depth, gyrification index, and fractal dimension. Red color means the significant increase in POI participants and blue color means the significant decrease in POI participants. L, left; R, right.

For further analysis, participants were stratified into two distinct age subgroups: the younger subgroup (ages 20–35 years, including 16 POI participants and 27 controls) and the older subgroup (ages 36–40 years, including 17 POI participants and 24 controls). The characteristics of the younger and older POI participants were nearly identical, except for parity (Tables S3‐S4). Our results showed that POI participants in the younger subgroup had significantly lower global GMV (one‐tailed *p* = 0.039), cortical thickness (*p* = 0.002), and sulcal depth (*p* = 0.022), and a higher gyrification index (*p* = 0.020) compared to controls (Figure S3). In contrast, no significant global differences were observed in the older subgroup. Furthermore, a greater number of brain regions exhibited significant decreases in GMV and cortical thickness in the younger subgroup (Figure 2), whereas the older subgroup showed a significant increase in GMV in the frontal lobe (Figure 3). Specifically, we observed a significant reduction in GMV in the younger POI participants compared with controls, particularly in the olfactory cortex and parahippocampal gyrus (Figure 2). In addition, significant decreases in cortical thickness were found in the bilateral frontal lobe, bilateral central lobe, left insula, and left transverse temporal gyrus among the younger POI participants compared to the controls (Figure 2). These findings suggest that earlier onset of POI is associated with more pronounced brain structural changes, particularly in regions related to memory and cognition. This highlights a potential age‐dependent vulnerability in brain structure among POI participants.

### 3.3 White matter structural network analysis

#### 3.3.1 Global properties of the structural network

No significant differences were observed in the MCC, CPL, and SW properties between the POI and control groups (Table S5). Consistent results were found in both subgroups (Tables S6 and S7). In this study, the structural networks of both POI participants and controls exhibited the typical small‐world topology, with MCC >1, CPL ≈1, and SW >1. No significant differences were found in the global network indicators between the POI and control groups.

#### 3.3.2 Changes of structural connectivity in POI participants

**FIGURE 4:**
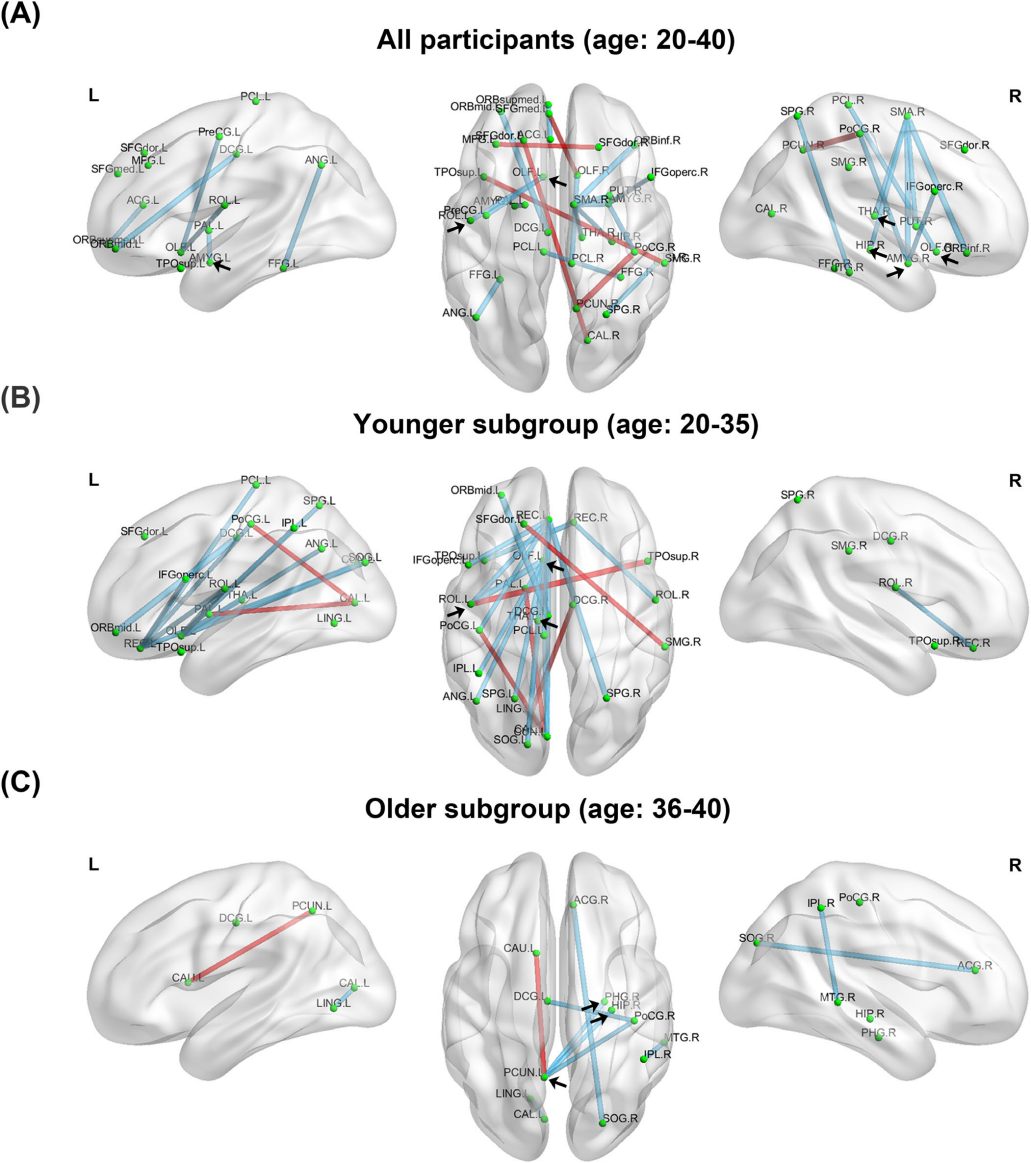
Significant difference in diffusion tensor imaging (DTI) structural connectivity between premature ovarian insufficiency (POI) and control in (A) all participants (ages 20–40 years), (B) younger subgroup (ages 20–35 years), (C) older subgroup (ages 36–40 years). A red edge means the connectivity is significantly increasing in POI participants and a blue edge means its connectivity is significantly decreasing in POI participants. Black arrows indicate the location of olfactory, thalamus, amygdala, hippocampus, parahippocampus, precuneus, and Rolandic operculum. The correspondence between abbreviations and brain regions was shown in Table S1. L, left; R, right.

In post hoc correlation analyses, we observed significantly reduced structural connections in POI participants compared to the control group, specifically in the bilateral olfactory cortex, bilateral amygdala, right hippocampus, and right thalamus (Figure 4A). In the younger subgroup (ages 20–35 years), significant reductions in connectivity were observed between the left olfactory cortex, left thalamus, and other brain regions (Figure 4B). Both the overall participant group (ages 20–40 years) and the younger subgroup showed a significant decline in connectivity between the left olfactory cortex and left Rolandic operculum. In the older subgroup (ages 36–40 years), connectivity between the left precuneus and the right hippocampus and right parahippocampal gyrus was significantly reduced in POI participants (Figure 4C). These findings suggest a reduction in the white matter fibers connecting these key regions to other areas. In addition to these reductions, some connectivities exhibited significant increases in POI participants, including edges connecting the superior frontal cortices, precuneus, postcentral gyrus, and superior temporal cortices (Figure 4A–C). These results point to a complex reorganization of structural connectivity in POI participants, characterized by both diminished and compensatory enhancements in specific brain regions.

#### 3.3.3 Changes in nodal features of structural networks in POI participants

**FIGURE 5:**
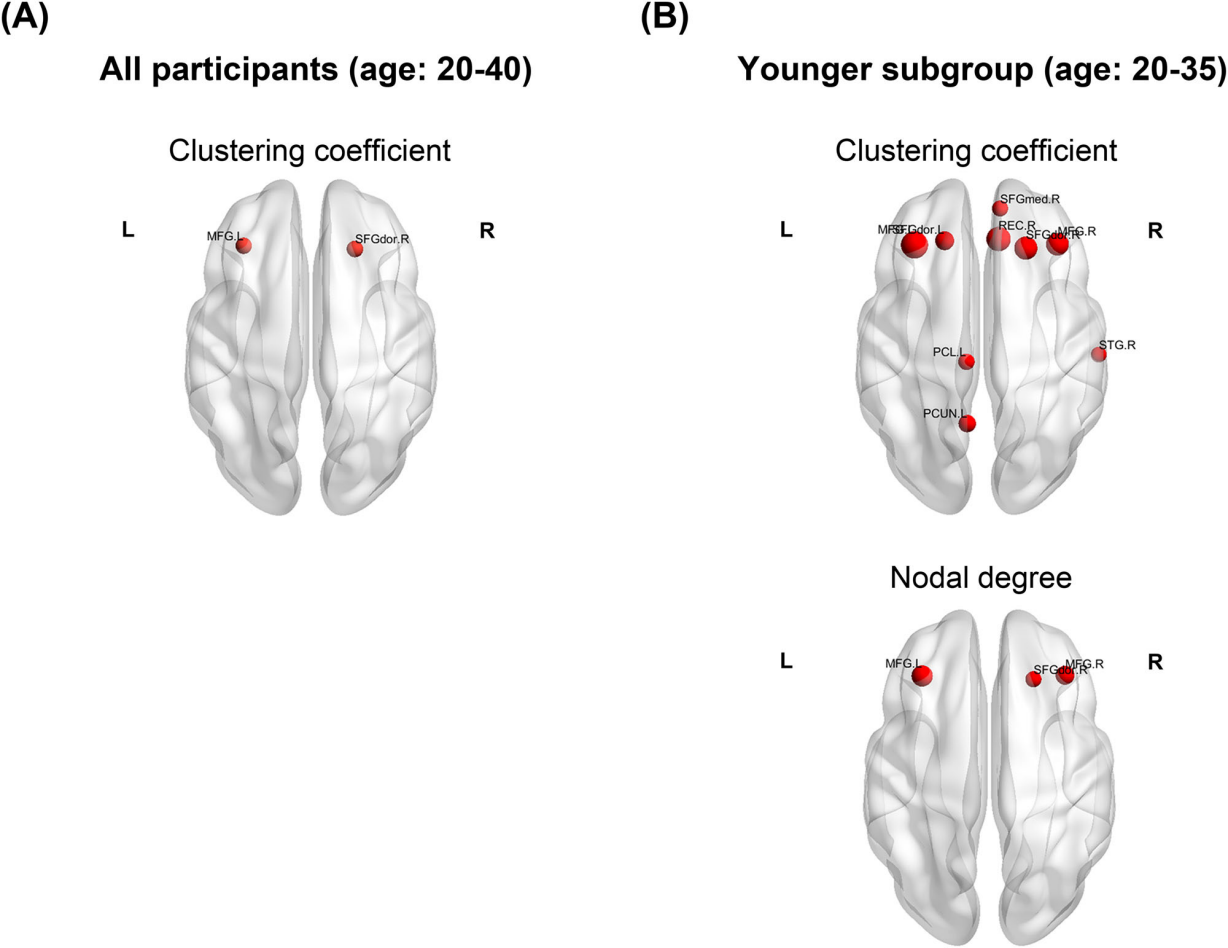
Significant difference in nodal features of diffusion tensor imaging (DTI) structural network. (A) Nodes with significant higher clustering coefficient in premature ovarian insufficiency (POI) participants (ages 20–40 years). (B) Nodes with higher clustering coefficient and nodal degree in POI participants within the younger subgroup (ages 20–35 years). The correspondence between abbreviations and brain regions was shown in Table S1. L, left; R, right.,

The analysis revealed a significant increase in the clustering coefficient within two regions of the frontal lobe in POI participants (Figure 5A), although no significant differences were observed in nodal degree or local efficiency. Further analysis showed a marked increase in the number of significantly heightened nodes in POI participants ages 20–35 years, accompanied by a significant rise in nodal degree within the frontal lobe and clustering coefficient within the frontal lobe, left paracentral lobe, left precuneus, and right superior temporal gyrus (Figure 5B), with no significant changes in local efficiency for this age group. However, no significant differences were found in clustering coefficient, nodal degree, or local efficiency between POI participants and control group in the older subgroup (ages 36–40 years). The increased clustering coefficient of nodes in the frontal lobe suggests a higher local centrality, forming clusters within these regions in POI participants. In addition, nodes in the frontal lobe showed an increase in degree centrality within the whole network when comparing younger POI participants to younger controls. These findings suggest that younger POI participants exhibit more pronounced alterations in brain network connectivity, particularly within the frontal lobe regions, indicating that the impact of POI on brain network structure may be more significant when it occurs at an earlier age.

### 3.4 Hippocampus and thalamus volumetric analysis

This study focused on the hippocampus and thalamus due to their critical roles in memory, learning, and sensory processing—functions commonly impaired in POI participants and related neurological conditions.^35^ Table 3 presents the statistical analysis results for the GMV of hippocampal and thalamic subfields between the POI group and the control group. The results indicate that POI participants exhibited significantly smaller GMV in the left subiculum (*p* = 0.026), right subiculum (*p* = 0.018), and right cornu ammonis 4 (CA4)/dentate gyrus (*p* = 0.050) compared to the control group. In addition, the GMV of the right sensory thalamus (*p* = 0.015), right posterior parietal thalamus (*p* = 0.006), left occipital thalamus (*p* = 0.042), and right lateral prefrontal thalamus (*p* = 0.020) was significantly smaller in the POI group compared to the control group. No significant differences were found in hippocampal subfields in either the younger or older subgroups. In younger POI participants, the GMV of the right sensory thalamus (*p* = 0.049) and right lateral prefrontal thalamus (*p* = 0.050) was reduced compared to controls in the same age group, whereas older POI participants exhibited decreased GMV in the right posterior parietal thalamus (*p* = 0.032) and left occipital thalamus (*p* = 0.045) compared to their controls (Table S8).

**TABLE 3:** Gray matter volume (cm^3^) of hippocampus and thalamus subfields in POI and control groups (ages 20–40 years).

Subfield with significant difference	POI group (*n *= 33)	Control group (*n *= 51)	*p*
Hippocampus			
Left subiculum	0.301 ± 0.022	0.313 ± 0.023	0.026^a^
Right subiculum	0.287 ± 0.021	0.300 ± 0.025	0.018^a^
Right CA4/dentate gyrus	0.613 ± 0.037	0.630 ± 0.038	0.050^a^
Thalamus			
Right sensory thalamus	0.149 ± 0.034	0.171 ± 0.043	0.015^a^
Right posterior parietal thalamus	0.818 ± 0.074	0.872 ± 0.091	0.006^a^
Left occipital thalamus	0.702 ± 0.069	0.733 ± 0.064	0.042^a^
Right lateral prefrontal thalamus	0.655 ± 0.085	0.710 ± 0.115	0.020^a^

*Note*: The data are shown as mean ± SD.

Abbreviations: CA4, cornu ammonis 4; POI, premature ovarian insufficiency; SD, standard deviation.

^a^ Student's *t‐*test for independent samples.

## 4 DISCUSSION

The association between POI and an increased risk of cognitive disorders, including AD, is well established.^36^ However, research investigating brain structural alterations and the underlying mechanisms in POI remains limited. This study presents the first evidence of significant gray matter morphology and white matter connectivity change in POI participants, despite the absence of clinical neurological deficits or overt cognitive decline. Specifically, POI participants exhibited reduced GMV and cortical thickness in critical regions involved in memory and emotional regulation, such as the parahippocampal gyrus and prefrontal cortex. Moreover, structural connectivity changes, assessed via DTI, were notably observed in sensory and executive function‐related regions, including the olfactory cortex, hippocampus, amygdala, thalamus, and frontal lobe. These findings suggest that the brain structural changes observed in POI participants may lay the foundation for future neurodegenerative alterations.

Early AD is characterized by gray matter atrophy in the medial temporal lobe, particularly the hippocampus and entorhinal cortex.^37^ Estrogen, a key neuroprotective hormone, is known to mitigate neurodegenerative processes and neuroinflammation.^38^, ^39^, ^40^, ^41^, ^42^, ^43^ Furthermore, research has shown that declining estrogen levels in women are associated with imaging endophenotypes indicative of an increased risk for AD.^44^ In our study, as expected, POI participants exhibited significantly lower estrogen levels and structural brain changes resembling early AD, including reduced GMV and cortical thickness in regions critical to AD‐related functions, underscoring the potential neurobiological impact of estrogen deficiency.^44^ Specifically, POI participants showed reduced GMV in the parahippocampal gyrus, a key region involved in memory and learning. Young POI participants (ages 20–35) also exhibited significant GMV reductions in the frontal, parietal, and temporal lobes, in line with findings in non‐human primates, which highlight estrogen's role in hippocampal‐dependent memory and prefrontal cortex function.^45^ In addition, our study revealed structural changes in the frontal and temporal lobes in POI participants, mirroring the decrease in estrogen's neuroprotective effects seen in women undergoing early menopause.^46^ Together, these results suggest that early estradiol loss may induce phenotypic brain changes that potentially increase the risk of dementia in later life.

Using DTI‐based tractography, we analyzed white matter fibers networks between brain regions. POI participants did not show significant changes in global graph‐theoretical features, suggesting that their structural connectivity did not undergo substantial global alterations up to the time of MRI assessment. Because structural connectivity is thought to reflect the substrate of functional brain patterns,^47^ this result aligns with the clinical absence of overt neurological disorders in the POI participants of this study. However, we observed significant reductions in the connectivity of specific brain regions, including the olfactory cortex, hippocampus, amygdala, and thalamus, with other areas. These changes align with key biomarkers of AD and mild cognitive impairment (MCI),^48^, ^49^, ^50^, ^51^, ^52^, ^53^ particularly as these regions are rich in estrogen receptors.^54^ This suggests that POI‐related estrogen deficiency may lead to brain alterations resembling those seen in AD and MCI, potentially increasing dementia risk.

Of interest, the structural network of POI participants also exhibited some significant increases in edges and nodes with higher network centrality. Most of the nodes with increased centrality or stronger connections were located in the frontal lobe. Previous studies have suggested a potential relationship between cortical neuronal loss and increased resting‐state functional connectivity in the rostral prefrontal cortex, which may be compensatory in nature, particularly in participants with MCI.^55^, ^56^ Similar compensatory processes have also been observed in white matter connectivity following brain injuries.^57^ We hypothesize that the increased clustering coefficient observed in two regions of the frontal lobe may serve as a compensation for early structural damage associated with hormonal disorders in POI women. However, this compensation likely depends on the integrity of the underlying brain structures. As estrogen levels decline further and neural damage progresses, the ability of these compensatory mechanisms to sustain normal function may diminish. This finding aligns with the “critical window hypothesis” for estrogen supplementation during perimenopause.^58^

Menopause syndrome, which includes symptoms such as mood changes, sleep disturbances, hot flashes, sweating, and memory decline, is thought to be linked to estrogen deficiency and its effects on brain function. In contrast to the typical menopausal age of around 50, women with POI experience these symptoms 10–30 years earlier. In this study, menopause symptoms in POI participants were assessed using the MENQOL. GMV reductions in the right parahippocampal gyrus, right subiculum, and CA4/dentate region were significantly negatively correlated with MENQOL scores, suggesting that lower GMV in these regions is associated with more severe menopause symptoms in POI participants. Among younger POI participants (ages 20–35), lower GMV in the temporal lobe and reduced cortical thickness in the frontal lobe were also correlated with higher MENQOL scores, indicating more pronounced effects of POI on brain function. In addition, increased nodal degree and clustering coefficient in the frontal and temporal regions showed significant negative correlations with MENQOL scores, further supporting the hypothesis of a compensatory mechanism in response to structural changes (Table S9). Moreover, reduced GMV and cortical thickness in younger POI participants were significantly negatively correlated with the time between the last menses and the diagnosis of POI, whereas no significant correlation was found in older participants (Table S10). These findings imply that an earlier onset of POI may lead to more substantial brain structural changes, highlighting the urgent need for interventions to prevent dementia in young POI participants. Despite the hormonal disruptions inherent to POI, no significant association was found between hormone levels and GMV or cortical thickness (Table S11), likely due to the small sample size. Furthermore, POI participants exhibited persistently elevated FSH levels exceeding 25 IU/L, indicating significant ovarian function decline. Emerging research suggests that FSH may act directly on hippocampal and cortical neurons to accelerate Aβ and tau deposition,^59^ warranting further investigation into its implications for AD risk in individuals with hormone imbalances resulting from ovarian dysfunction.

In addition to neurodegeneration, vascular factors likely play a critical role in brain health among women with POI. Estrogen deficiency, a hallmark of POI, not only disrupts neuroprotective mechanisms but also negatively impacts vascular health, thereby increasing the risk of cardiovascular diseases and strokes.^12^ These vascular changes may contribute to the observed structural brain alterations, particularly in the hippocampus and thalamus, both of which are highly sensitive to hormonal and vascular insults.^60^, ^61^ Moreover, apolipoprotein E (*APOE*) ε4 carriers, known to have estrogen‐binding sites, exhibit pronounced atrophy in specific regions, including the thalamus and hippocampus.^54^, ^62^ Previous research has shown that both AD and MCI groups exhibit significantly smaller hippocampal volumes compared to controls, including areas like the CA4, dentate gyrus, and subiculum.^63^, ^64^ In AD patients, volume changes in the right thalamus were significantly associated with episodic memory, learning, and semantic processing.^65^ Similarly, in our study, we found that GMV in the bilateral subiculum and right CA4/dentate gyrus was significantly reduced in POI group. In addition, within the thalamus, POI participants showed a decline in GMV in the sensory, posterior parietal, and lateral prefrontal subregions of the right hemisphere, as well as in the left occipital thalamus. These findings may represent early brain changes associated with cognitive function in POI patients, potentially preceding more significant cognitive decline.

In contrast to previous studies,^13^ our study offers several novel contributions. It is the first to investigate potential brain changes in newly diagnosed POI patients with an unknown cause. As discussed earlier, we identified abnormal brain structural changes in POI women at both the region‐ and network‐levels, which may help clarify the neurological mechanisms underlying clinical symptoms and neurobiological alterations in these women. Second, although we observed brain structural changes in POI participants that resemble those seen in early AD, we emphasize that these changes are not limited to AD‐related regions. Our findings suggest that the progression of POI may increase the risk of dementia, not only in AD but also in vascular dementia and other subtypes.

However, our study has several limitations. The relatively small sample size calls for validation in larger cohorts. In addition, as this is a cross‐sectional study, longitudinal follow‐up is necessary to confirm whether the observed structural changes progress over time and are linked to cognitive decline. Finally, due to the exploratory nature of this study, we did not adjust for multiple comparisons. Future studies will also incorporate more comprehensive neuropsychological evaluations to explore subtle cognitive and neurological changes.

In conclusion, POI participants exhibit abnormalities in brain regions associated with memory, emotional regulation, and executive function, including the hippocampus, frontal lobe, thalamus, olfactory cortex, and amygdala. Notably, younger participants demonstrate more pronounced brain structural changes. These alterations resemble those in early dementia, including AD, but are not limited to AD‐specific regions. Given the exploratory nature of this study and its small sample size, further research involving larger, longitudinal cohorts is essential to validate these findings. Nonetheless, our results underscore the urgent need for early interventions addressing both hormonal imbalances and vascular health to mitigate the long‐term risks of cognitive impairment and dementia in women with POI.

## AUTHOR CONTRIBUTIONS

Dongmei Lai, Puming Zhang, and Zhaoxia Qian were responsible for the conception and design of the study. Yu Zhang and Taotao Sun performed the magnetic resonance imaging (MRI) scan. Shuang Yuan, Yuchen Gong, Yu Zhang, and Wenjiao Cao collected, analyzed, and interpreted the data and wrote the original draft. Taotao Sun, Liutong Wei, Junyan Sun, Lulu Wang, Yu Wei, Qiuwan Zhang, and Qian Wang organized and supervised data collection and analyzed the data. Dongmei Lai and Puming Zhang played a key role in conceptualization, methodology, review, and editing. All authors approved the final version of the manuscript.

## CONFLICT OF INTEREST STATEMENT

The authors report no conflicts of interest.

## CONSENT STATEMENT

All participants provided written informed consent.

## Supporting information



Supporting Information

Supporting Information
