# Supplementary material for: Brain structural alterations in young women with premature ovarian insufficiency: Implications for dementia risk
Source: Alzheimers Dement. 2025 Mar 27;21(3):e70111. doi: 10.1002/alz.70111 (PMC11947759; doi:10.1002/alz.70111)
Supplement: Supplementary file 2 — Supporting Information [file ALZ-21-e70111-s002.docx]

Supplementary Table 1. Cerebral regions defined in Anatomical Automatic Labeling (AAL) atlas.

| Brain region | Left hemisphere | | Right hemisphere | | Brain region | Left hemisphere | | Right hemisphere | |
| --- | --- | --- | --- | --- | --- | --- | --- | --- | --- |
|  | No. | Abbrev. | No. | Abbrev. |  | No. | Abbrev. | No. | Abbrev. |
| Precental gyrus | 1 | PreCG.L | 2 | PreCG.R | Lingual gyrus | 47 | LING.L | 48 | LING.R |
| Superior frontal gyrus, dorsolateral | 3 | SFGdor.L | 4 | SFGdor.R | Superior occipital gyrus | 49 | SOG.L | 50 | SOG.R |
| Superior frontal gyrus, orbital part | 5 | ORBsup.L | 6 | ORBsup.R | Middle occipital gyrus | 51 | MOG.L | 52 | MOG.R |
| Middle frontal gyrus | 7 | MFG.L | 8 | MFG.R | Inferior occipital gyrus | 53 | IOG.L | 54 | IOG.R |
| Middle frontal gyrus, orbital part | 9 | ORBmid.L | 10 | ORBmid.R | Fusiform gyrus | 55 | FFG.L | 56 | FFG.R |
| Inferior frontal gyrus, opercular part | 11 | IFGoperc.L | 12 | IFGoperc.R | Postcentral gyrus | 57 | PoCG.L | 58 | PoCG.R |
| Inferior frontal gyrus, triangular part | 13 | IFGtriang.L | 14 | IFGtriang.R | Superior parietal gyrus | 59 | SPG.L | 60 | SPG.R |
| Inferior frontal gyrus, orbital part | 15 | ORBinf.L | 16 | ORBinf.R | Inferior parietal, but supramarginal and angular gyri | 61 | IPL.L | 62 | IPL.R |
| Rolandic operculum | 17 | ROL.L | 18 | ROL.R | Supramarginal gyrus | 63 | SMG.L | 64 | SMG.R |
| Supplementary motor area | 19 | SMA.L | 20 | SMA.R | Angular gyrus | 65 | ANG.L | 66 | ANG.R |
| Olfactory cortex | 21 | OLF.L | 22 | OLF.R | Precuneus | 67 | PCUN.L | 68 | PCUN.R |
| Superior frontal gyrus, medial | 23 | SFGmed.L | 24 | SFGmed.R | Paracentral lobule | 69 | PCL.L | 70 | PCL.R |
| Superior frontal gyrus, medial orbital | 25 | ORBsupmed.L | 26 | ORBsupmed.R | Caudate nucleus | 71 | CAU.L | 72 | CAU.R |
| Gyrus rectus | 27 | REC.L | 28 | REC.R | Lenticular nucleus, putamen | 73 | PUT.L | 74 | PUT.R |
| Insula | 29 | INS.L | 30 | INS.R | Lenticular nucleus, pallidum | 75 | PAL.L | 76 | PAL.R |
| Anterior cingulate and paracingulate gyri | 31 | ACG.L | 32 | ACG.R | Thalamus | 77 | THA.L | 78 | THA.R |
| Median cingulate and paracingulate gyri | 33 | DCG.L | 34 | DCG.R | Heschl gyrus | 79 | HES.L | 80 | HES.R |
| Posterior cingulate gyrus | 35 | PCG.L | 36 | PCG.R | Superior temporal gyrus | 81 | STG.L | 82 | STG.R |
| Hippocampus | 37 | HIP.L | 38 | HIP.R | Temporal pole: superior temporal gyrus | 83 | TPOsup.L | 84 | TPOsup.R |
| Parahippocampal gyrus | 39 | PHG.L | 40 | PHG.R | Middle temporal gyrus | 85 | MTG.L | 86 | MTG.R |
| Amygdala | 41 | AMYG.L | 42 | AMYG.R | Temporal pole: middle temporal gyrus | 87 | TPOmid.L | 88 | TPOmid.R |
| Calcarine fissure and surrounding cortex | 43 | CAL.L | 44 | CAL.R | Inferior temporal gyrus | 89 | ITG.L | 90 | ITG.R |
| Cuneus | 45 | CUN.L | 46 | CUN.R |  |  |  |  |  |

Supplementary Table 2. Clinical scale assessments of POI and control participants.

| Scales | POI group (n=33) | Control group (n=51) | P |
| --- | --- | --- | --- |
| MMSE | 29.4±0.9 | 29.4±1.1 | 0.786^b^ |
| PHQ-9 | 6.2±6.1 | 4.2±4.3 | 0.054^b^ |
| SAS | 41.7±10.5 | 37.9±8.5 | 0.097^b^ |
| MENQOL scores |  |  |  |
| Total items | 12.3±4.9 | 7.4±2.7 | <0.001^a^ |
| Vasomotor items | 3.1±2.1 | 1.4±0.8 | <0.001^b^ |
| Psychosocial items | 3.5±1.4 | 2.6±1.3 | 0.003^b^ |
| Physical items | 2.8±1.2 | 2.1±0.8 | 0.003^a^ |
| Sexual items | 2.9±1.8 | 1.3±0.7 | <0.001^b^ |

Abbreviations: POI, premature ovarian insufficiency; MMSE, Mini-Mental State Examination; PHQ-9, Patient Health Questionnaire-9; SAS, Self-Rating Anxiety Scale; MENQOL, Menopause-Specific Quality of Life Questionnaire.

The date was shown as Mean±SD.

^a^ Student’s t-test for independent samples.

^b^ Mann-Whitney U-test.

Supplementary Table 3. Demographic and clinical characteristics of younger and older POI participants.

| Characteristic | younger POI (n=16) | older POI (n=17) | P |
| --- | --- | --- | --- |
| Age (y) | 29.9±4.4 | 38.4±1.4 | <0.001^a^ |
| BMI (kg/m^2^) | 20.7±2.4 | 21.1±2.4 | 0.646^a^ |
| Age at menarche (y) | 13.1±1.1 | 13.4±1.5 | 0.465^a^ |
| Parity (time) |  |  | 0.019^b^ |
| 0 | 13 (81.2) | 7 (41.2) |  |
| ≥1 | 3 (18.8) | 10 (58.8) |  |
| Smoking status |  |  | 0.295^b^ |
| No | 15 (93.8) | 17 (100.0) |  |
| Yes | 1 (6.2) | 0 (0.0) |  |
| Alcohol consumption |  |  | 0.849^b^ |
| No | 7 (43.8) | 8 (47.1) |  |
| Yes | 9 (56.2) | 9 (52.9) |  |
| ALT (U/L) | 17.9±7.8 | 19.2±8.8 | 0.709^c^ |
| AST (U/L) | 19.9±4.7 | 19.5±7.9 | 0.859^a^ |
| Creatinine (umol/L) | 62.4±9.1 | 65.9±6.9 | 0.220^a^ |
| FSH (IU/L) | 60.2±25.2 | 71.9±27.4 | 0.213^a^ |
| LH (IU/L) | 36.0±21.5 | 32.8±12.8 | 0.986^c^ |
| E2 (pmol/L) | 217.1±336.2 | 129.3±130.6 | 0.557^c^ |
| T (nmol/L) | 1.6±0.8 | 1.2±0.7 | 0.168^c^ |
| PRL (ug/L) | 11.3±6.9 | 8.0±3.1 | 0.136^c^ |
| AMH (ng/mL) | 0.1±0.0 | 0.1±0.0 | 0.817^c^ |

Abbreviations: POI, premature ovarian insufficiency; BMI, body mass index; ALT, Alanine aminotransferase; AST, Spartate aminotransferase; FSH, follicle-stimulating hormone; LH, luteinizing hormone; E2, estradiol; T, testosterone; PRL, prolactin; AMH, anti-Mullerian hormone; y, years.

The date was shown as Mean±SD or count (percentage).

*(Time) after parity refers to the number of childbirths (parity).

^a^ Student’s t-test for independent samples.

^b^ Chi-square test.

^c^ Mann-Whitney U-test.

Supplementary Table 4. Clinical scale assessments of younger and older POI participants.

| Scales | younger POI (n=16) | older POI (n=17) | P |
| --- | --- | --- | --- |
| MMSE | 29.7±0.6 | 29.2±1.1 | 0.231^b^ |
| PHQ-9 | 4.4±3.1 | 8.0±7.6 | 0.127^b^ |
| SAS | 39.3±9.5 | 44.0±11.1 | 0.204^b^ |
| MENQOL scores |  |  |  |
| Total items | 12.0±5.3 | 12.7±4.7 | 0.683^a^ |
| Vasomotor items | 3.1±1.8 | 3.2±2.3 | 0.986^b^ |
| Psychosocial items | 3.4±1.6 | 3.6±1.2 | 0.754^a^ |
| Physical items | 2.5±1.1 | 3.2±1.2 | 0.095^a^ |
| Sexual items | 3.0±2.1 | 2.8±1.5 | 0.901^b^ |

Abbreviations: POI, premature ovarian insufficiency; MMSE, Mini-Mental State Examination; PHQ-9, Patient Health Questionnaire-9; SAS, Self-Rating Anxiety Scale; MENQOL, Menopause-Specific Quality of Life Questionnaire.

The date was shown as Mean±SD.

^a^ Student’s t-test for independent samples.

^b^ Mann-Whitney U-test.

Supplementary Table 5. Global properties of structural networks in POI group and control group (age of 20-40 years old).

| Characteristic | POI group (n=33) | Control group (n=51) | P |
| --- | --- | --- | --- |
| Mean clustering coefficient | 1.241±0.142 | 1.244±0.102 | 0.595^a^ |
| Characteristic path length | 1.008±0.012 | 1.010±0.014 | 0.905^a^ |
| Small-worldness | 1.231±0.138 | 1.232±0.099 | 0.564^a^ |

Abbreviations: POI, premature ovarian insufficiency.

The date was shown as Mean±SD.

^a^ Student’s t-test for independent samples.

Supplementary Table 6. Global properties of structural networks in POI and control participants of the younger subgroup (age of 20-35 years old).

| Characteristics | POI group (n=16) | Control group (n=27) | P |
| --- | --- | --- | --- |
| Mean clustering coefficient | 1.245±0.160 | 1.245±0.102 | 0.643^a^ |
| Characteristic path length | 1.011±0.015 | 1.010±0.014 | 0.321^a^ |
| Small-worldness | 1.231±0.156 | 1.232±0.099 | 0.503^a^ |

Abbreviations: POI, premature ovarian insufficiency.

The date was shown as Mean±SD.

^a^ Student’s t-test for independent samples.

Supplementary Table 7. Global properties of structural networks in POI and control participants of the older subgroup (age of 36-40 years old).

| Characteristics | POI group (n=17) | Control group (n=24) | P |
| --- | --- | --- | --- |
| Mean clustering coefficient | 1.237±0.130 | 1.225±0.093 | 0.947^a^ |
| Characteristic path length | 1.005±0.009 | 1.006±0.010 | 0.905^a^ |
| Small-worldness | 1.230±0.123 | 1.217±0.088 | 0.947^a^ |

Abbreviations: POI, premature ovarian insufficiency.

The date was shown as Mean±SD.

^a^ Student’s t-test for independent samples.

Supplementary Table 8. Gray matter volume (cm^3^) of thalamus subfields in POI and control participants in the younger subgroup (age of 20-35 years old) and the older subgroup (age of 36-40 years old).

| Subfield in thalamus with significant difference | POI group (n=33) | Control group (n=51) | P |
| --- | --- | --- | --- |
| Younger subgroup | POI group (n=16) | Control group (n=27) |  |
| Right sensony thalamus | 0.144±0.027 | 0.169±0.044 | 0.049^a^ |
| Right lateral prefrontal thalamus | 0.655±0.086 | 0.724±0.119 | 0.050^a^ |
| Older subgroup | POI group (n=17) | Control group (n=24) |  |
| Right posterior parietal thalamus | 0.806±0.078 | 0.862±0.080 | 0.032^a^ |
| Left occipital thalamus | 0.681±0.075 | 0.725±0.059 | 0.045^a^ |

Abbreviations: POI, premature ovarian insufficiency.

The date was shown as Mean±SD.

^a^ Student’s t-test for independent samples.

Supplementary Table 9. The relationship between significantly changed features in POI participants and their menopause-related symptoms.

| POI Group | Feature | T-value with controls^a^ | P-value (difference with controls) | MENQOL score | Correlation with MENQOL score^b^ | P-value (correlated with MENQOL score) |
| --- | --- | --- | --- | --- | --- | --- |
| Age of 20-40 | GMV of right parahippocampal gyrus | -1.913 | 0.030* | physical | -0.385 | 0.027 |
|  | Gyrification index of left superior parietal lobe | 2.385 | 0.019 | psychosocial | -0.356 | 0.042 |
|  | GMV of right subiculum | -2.406 | 0.018 | physical | -0.459 | 0.007 |
|  | GMV of right CA4/dentate gyrus | -1.990 | 0.050 | physical | -0.428 | 0.013 |
| Age of 20-35 | GMV of right middle temporal gyrus | -2.129 | 0.039 | physical | -0.535 | 0.033 |
|  | Cortical thickness of left inferior parietal lobe | -3.612 | 0.001 | psychosocial | 0.582 | 0.018 |
|  | Cortical thickness of left superior parietal lobe | -3.085 | 0.004 | psychosocial | 0.706 | 0.002 |
|  |  |  |  | physical | 0.564 | 0.023 |
|  |  |  |  | total | 0.534 | 0.033 |
|  | Cortical thickness of right inferior parietal lobe | -2.592 | 0.013 | psychosocial | 0.569 | 0.021 |
|  | Cortical thickness of right lateral orbitofrontal lobe | -2.502 | 0.016 | sexual | -0.555 | 0.026 |
|  | Cortical thickness of right superior parietal lobe | -3.431 | 0.001 | psychosocial | 0.645 | 0.007 |
|  | Sucal depth of right postcentral lobe | -2.644 | 0.012 | psychosocial | -0.520 | 0.039 |
|  | Node degree of left frontal middle gyrus | 2.651 | 0.008 | physical | -0.517 | 0.040 |
|  | Node degree of right frontal middle gyrus | 2.349 | 0.019 | physical | -0.609 | 0.012 |
|  |  |  |  | sexual | -0.579 | 0.019 |
|  |  |  |  | total | -0.625 | 0.010 |
|  | Clustering coefficient of left frontal superior gyrus | 2.349 | 0.019 | sexual | -0.680 | 0.004 |
|  |  |  |  | total | -0.526 | 0.036 |
|  | Clustering coefficient of left frontal middle gyrus | 3.379 | 0.001 | physical | -0.619 | 0.011 |
|  |  |  |  | sexual | -0.718 | 0.002 |
|  |  |  |  | total | -0.665 | 0.005 |
|  | Clustering coefficient of right frontal superior medial gyrus | 1.998 | 0.046 | sexual | -0.515 | 0.041 |
|  |  |  |  | total | -0.522 | 0.038 |
|  | Clustering coefficient of right rectus gyrus | 3.028 | 0.002 | physical | -0.611 | 0.012 |
|  |  |  |  | sexual | -0.545 | 0.029 |
|  |  |  |  | total | -0.581 | 0.018 |
|  | Clustering coefficient of right temporal superior gyrus | 1.972 | 0.049 | sexual | -0.516 | 0.041 |
| Age of 36-40 | Gyrification index of right middle temporal lobe | -2.130 | 0.040 | psychosocial | -0.518 | 0.033 |
|  |  |  |  | sexual | -0.504 | 0.039 |
|  |  |  |  | total | -0.564 | 0.018 |
|  | Fractal dimension of right paracentral lobe | -2.073 | 0.045 | physical | -0.421 | 0.034 |

*Indicates one-tailed analysis; all others are two-tailed.

^a^ Student’s t-test for independent samples.

^b^ Pearson's correlation analysis.

Supplementary Table 10. Associations between the duration from last menses to POI diagnosis and changes in gray matter volume and cortical thickness.

| POI Group | Feature | T-value with controls^a^ | P-value (difference with controls) | Correlation with duration^b^ | P-value (correlated with duration) |
| --- | --- | --- | --- | --- | --- |
| Age of 20-40 | GMV of right lateral prefrontal thalamus | -2.376 | 0.020 | -0.404 | 0.020 |
| Age of 20-35 | Global cortical thickness | -3.234 | 0.002 | -0.433 | 0.047* |
|  | GMV of left precentral | -2.279 | 0.028 | -0.514 | 0.042 |
|  | cortical thickness of left precentral lobe | -3.185 | 0.003 | -0.603 | 0.013 |
|  | cortical thickness of left superior parietal lobe | -3.085 | 0.004 | -0.537 | 0.032 |
|  | cortical thickness of right precentral lobe | -2.418 | 0.020 | -0.589 | 0.016 |
|  | cortical thickness of right superior parietal lobe | -3.431 | 0.001 | -0.532 | 0.034 |
|  | GMV of right sensory thalamus | -2.028 | 0.049 | -0.629 | 0.009 |
| Age of 36-40 | None |  |  |  |  |

* Indicates one-tailed analysis; all others are two-tailed.

^a^ Student’s t-test for independent samples.

^b^ Pearson's correlation analysis.

Supplementary Table 11. Associations of reproductive hormones to gray matter volume and cortical thickness.

|  | Total (n=84) | | POI group (n=33) | | Control group (n=51) | |
| --- | --- | --- | --- | --- | --- | --- |
|  | GMV | CT | GMV | CT | GMV | CT |
|  | β(SE) | β(SE) | β(SE) | β(SE) | β(SE) | β(SE) |
| FSH | 0.104 (0.144) | 0.000 (0.000) | 0.097 (0.266) | 0.001 (0.001) | -1.685 (3.428) | -0.004 (0.007) |
| LH | 0.119 (0.280) | 0.000 (0.001) | 0.024 (0.394) | 0.001 (0.001) | -0.081 (0.939) | -0.002 (0.002) |
| E2 | -0.003 (0.014) | 0.000 (0.000) | -0.013 (0.028) | 0.000 (0.000) | 0.001 (0.017) | 0.000 (0.000) |
| T | 9.925 (8.727) | -0.002 (0.018) | 14.535 (9.256) | 0.033 (0.021) | 3.818 (16.120) | -0.068 (0.029) |
| PRL | -1.006 (0.812) | -0.002 (0.002) | 0.187 (1.371) | -0.002 (0.003) | -1.208 (1.105) | -0.003 (0.002) |
| AMH | 0.418 (1.789) | 0.003 (0.004) | -646.665 (781.105) | -1.601 (1.728) | 0.795 (2.590) | -0.001 (0.005) |

Abbreviations: POI, premature ovarian insufficiency; BMI, body mass index; FSH, follicle-stimulating hormone; LH, luteinizing hormone; E2, estradiol; T, testosterone; PRL, prolactin; AMH, anti-Mullerian hormone; GMV, gray matter volume; CT, cortical thickness.

Covariates: age, BMI, smoking, parity, smoking status, alcohol consumption.

Supplementary Figure 1. The time between last menses and diagnosis of POI. The average time was 3.4±1.6 months (Mean ± SD).

Abbreviations: POI, premature ovarian insufficiency.

Supplementary Figure 2. Menopausal Quality of Life (MENQOL) (Chinese and English version) questionnaire.


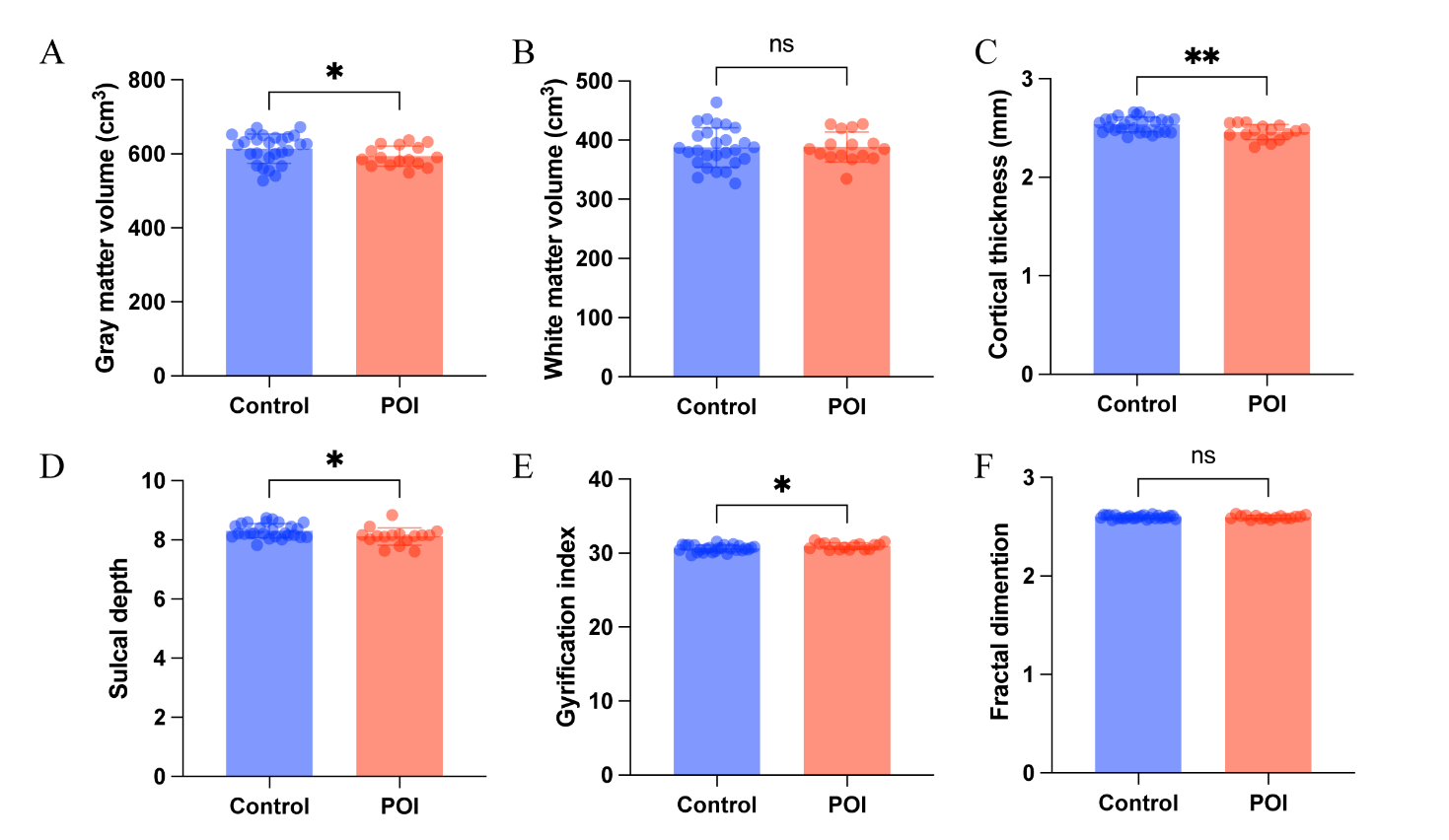


Supplementary Figure 3. Global voxel-based morphometry and surface-based morphometry features between POI and control participants in the younger subgroup (age of 20-35 years old). (A) gray matter volume, (B) white matter volume, (C) cortical thickness, (D) sulcal depth, (E) gyrification index, (F) fractal dimention. *P<0.05, ** P<0.01. POI, premature ovarian insufficiency
